# Editorial: Anatomy and Plasticity in Large-Scale Brain Models

**DOI:** 10.3389/fnana.2016.00108

**Published:** 2016-11-07

**Authors:** Markus Butz, Wolfram Schenck, Arjen van Ooyen

**Affiliations:** ^1^Simulation Laboratory Neuroscience, Bernstein Facility for Simulation and Database Technology, Institute for Advanced Simulation, Jülich Aachen Research Alliance, Jülich Research CenterJülich, Germany; ^2^Faculty of Engineering and Mathematics, Bielefeld University of Applied SciencesBielefeld, Germany; ^3^Department of Integrative Neurophysiology, VU University AmsterdamAmsterdam, Netherlands

**Keywords:** brain models, simulation, supercomputing, high-performance computing, anatomy, connectivity, structural plasticity

## Introduction

Supercomputing facilities are becoming increasingly available for simulating electrical activity in large-scale neuronal networks. On today's most advanced supercomputers, networks with up to a billion of neurons can be readily simulated. However, building biologically realistic, full-scale brain models requires more than just a huge number of neurons. In addition to network size, the detailed local and global anatomy of neuronal connections is of crucial importance. Moreover, anatomical connectivity is not fixed, but can rewire throughout life (structural plasticity; Butz et al., 2009)—an aspect that is missing in most current network models, in which plasticity is confined to changes in synaptic strength (synaptic plasticity).

The papers in this research topic, which may broadly be divided into three themes, aim to bring together high-performance computing with recent experimental and computational research in neuroanatomy. In the first theme (fiber connectivity), new methods are described for measuring and data-basing microscopic and macroscopic connectivity. In the second theme (structural plasticity), novel models are introduced that incorporate morphological plasticity and rewiring of anatomical connections. In the third theme (large-scale simulations), simulations of large-scale neuronal networks are presented with an emphasis on anatomical detail and plasticity mechanisms. Together, the papers in this research topic contribute to extending high-performance computing in neuroscience to encompass anatomical detail and plasticity.

## Fiber connectivity

Investigating the brain's connectivity requires multiscale approaches and hence strategies for integrating data across different spatial scales. Axer et al. demonstrate how to bridge microscopic visualizations of fibers obtained by 3D-PLI (polarized light imaging; Axer et al., 2011) to meso- or macro-scopic fiber orientations based on dMRI (diffusion magnetic resonance imaging). A relatively new technique, 3D-PLI is applicable to microtome sections of postmortem brains and uses birefringence of brain tissue, induced by optical anisotropy of the myelin sheaths around axons, to derive a 3D description of the underlying fiber architecture. To be able to link 3D-PLI to dMRI measurements, the authors introduce fiber orientation distribution functions (ODFs) extracted from 3D-PLI. They demonstrate the validity of their approach with simulated 3D-PLI data as well as real 3D-PLI data from the human brain and the brain of a hooded seal.

Capturing different aspects of brain organization, such as connectivity and molecular composition, necessitates the use of different neuroimaging techniques. To subsequently integrate the multiscale and multimodal data into a complete 3D brain model requires an accurate definition of the spatial positions of structural entities. Defined by MRI, the Waxholm Space (WHS) () provides such a reference space for rodent brain data. The aim of the study by Schubert et al. was to extend the WHS rat brain atlas with information about cytoarchitecture, receptor expression and spatial orientation of fiber tracts, derived from autoradiography and PLI images. To incorporate these distinct classes of information into the WHS, the authors improved currently available registration algorithms to align sections and to correct for deformations. The extended WHS rat brain atlas now enables combined studies on receptor and cell distributions as well as fiber densities in the same anatomical structures at microscopic scales. Furthermore, the methods developed facilitate future integration of data of other modalities.

## Structural plasticity

According to the long-standing connectionists' dogma, information is stored in the connection weights of neural networks. However, in biological neural networks, a synaptic connection is much more than just a weight factor, and a wealth of biological mechanisms can bring about changes in functional and structural connectivity. The following papers deal with models that go beyond the traditional modeling concept of plasticity as merely up- or down-regulating synaptic connection strength (synaptic plasticity).

Fauth and Tetzlaff review experimental and modeling studies that address the formation and deletion of synapses (structural plasticity) and how this is regulated by electrical activity. Whereas adapting existing synapses in a Hebbian manner predominantly serves memory consolidation, the *de novo* formation of synapses and deletion of existing synapses can, together with other synaptic mechanisms, grant stability to a network facing continuously changing inputs. The authors point out that these different plasticity mechanisms may be involved in different neural functions and, remarkably, may respond very differently to changes in neuronal activity.

Changes in the anatomical layout of connections (structural plasticity) are crucially dependent on morphological changes in individual neurons. A lack of understanding of how structural changes affect network function is partly due to the absence of tools for studying the impact of neuronal morphology on neuronal function. Therefore, Bezelos et al. introduce a simulation tool for systematically varying the morphology of any type of neuron, which will help investigate the role of neuronal morphology and morphological changes in large-scale neuronal networks.

Diaz-Pier et al. go beyond the level of single neurons and describe an approach based on a recent model of homeostatic structural plasticity (Butz and van Ooyen, 2013), which for the first time enables growing from scratch the neuronal network connectivity of a cortical column *in silico*. The resulting connectivity shows remarkable similarities with a real cortical column.

In biologically realistic, sparsely connected cortical networks, Knoblauch and Sommer study what the computational contribution of structural plasticity is to memory formation based on synaptic plasticity. They show that neuronal networks with structural plasticity can more efficiently adapt their connectivity to the computational problem at hand. As a consequence, structural plasticity may significantly increase the number of stably maintained memory items and may even account for psychological phenomena such as the spacing effect in rehearsal learning (Greene, 1989).

## Large-scale simulations

The third theme concerns large-scale simulations, with a special focus on anatomic detail and/or plasticity mechanisms. Because of the high computational demands of such simulations, it is crucial to have the right hardware and software tools available. Breit et al., Gosui and Yamazaki, and Knight et al. report on recent developments in this area. Furthermore, Zhou et al. show how to successfully employ established neuro-simulators for this purpose, and Kozloski how to move up to full brain-scale models.

Breit et al. developed the novel simulation framework NeuroBox and applied it to the investigation of the interplay between synapse loss and signaling synchrony. NeuroBox provides a link between the description of the morphology of neurons and networks on the one hand and the simulation of such networks in the simulation framework UG4 (Vogel et al., 2013) on the other hand. This combination enables the simulation of anatomically detailed models of large networks on supercomputers with good scaling behavior.

Gosui and Yamazaki tackle the problem of how to carry out long-term simulations within a manageable time frame. They studied the long-term gain adaptation of optokinetic response eye movements over a period of 5 days of simulated time in a large-scale cerebellar model. By implementing the simulation software on highly parallel processors (graphics processing units; GPUs), the simulation could be carried out in real-world time. This approach opens up new opportunities for studying long-term plasticity in neural networks, e.g., in the context of memory consolidation.

Knight et al. demonstrate that large-scale models that incorporate complex and demanding plasticity mechanisms (in this case, based on the Bayesian Confidence Propagation Neural Network paradigm; Lansner and Holst, 1996) can be simulated in a highly power-efficient way on the SpiNNaker neuromorphic architecture. Furthermore, in this study the largest plastic neural network ever was simulated on neuromorphic hardware. This shows that (some) neuromorphic hardware is already up to the task of simulating detailed anatomy and plasticity in large-scale brain models.

Zhou et al. rely in their work on the established neuro-simulator NEURON (Hines and Carnevale, 2001). They implemented a large-scale model of the olfactory bulb that includes plasticity mechanisms. They investigated how prior odor experience influences the representation of new odor inputs. The results show that prior experience changes the pattern in which sparse responses occur in different sub-networks within the olfactory bulb. From a methodological point of view, this study demonstrates how to set up a large-scale simulation of a plastic neural network with rather detailed anatomy.

Kozloski positions his modeling and simulation work at brain-scale, comprising the neocortex, basal ganglia and thalamus. The proposed model is based on the principle of information-based exchange and does not cover anatomical details. Nevertheless, it is grounded in neuroanatomical observations and accounts for various forms of plasticity. Modeling at this rather abstract level enables long-term simulations at brain-scale and extensive parameter variations. In this specific study, variations in dynamic set points and modulations were investigated, leading to a theory about the emergence of neurodegenerative diseases.

## Conclusion

Biologically realistic large-scale brain models require, besides a huge number of neurons, the right layout of local and global connections. Moreover, they need to capture the plasticity mechanisms operative in the adult brain. These mechanisms include, in addition to changes in the strength of synapses, structural modifications in anatomical connections. With the articles in this research topic, we hope that the reader will become aware of the methods and models by which large-scale brain networks running on supercomputing facilities may be extended to include anatomical detail and the full plastic potential of the brain.

## Author contributions

MB, WS, and AvO wrote the Editorial.

### Conflict of interest statement

The authors declare that the research was conducted in the absence of any commercial or financial relationships that could be construed as a potential conflict of interest.
